# Green tea (*Camellia sinensis*) and cancer prevention: a systematic review of randomized trials and epidemiological studies

**DOI:** 10.1186/1749-8546-3-12

**Published:** 2008-10-22

**Authors:** Jianping Liu, Jianmin Xing, Yutong Fei

**Affiliations:** 1 Centre for Evidence-Based Chinese Medicine, Beijing University of Chinese Medicine, Beijing 100029, PR China; 2 National Research Centre in Complementary and Alternative Medicine (NAFKAM), University of Tromso, Norway; 3 Division of Chinese Medicine, RMIT University, Melbourne, Australia

## Abstract

**Background:**

Green tea is one of the most popular beverages worldwide. This review summarizes the beneficial effects of green tea on cancer prevention.

**Methods:**

Electronic databases, including PubMed (1966–2008), the Cochrane Library (Issue 1, 2008) and Chinese Biomedical Database (1978–2008) with supplement of relevant websites, were searched. There was no language restriction. The searches ended at March 2008. We included randomized and non-randomized clinical trials, epidemiological studies (cohort and case-control) and a meta-analysis. We excluded case series, case reports, *in vitro *and animal studies. Outcomes were measured with estimation of relative risk, hazard or odd ratios, with 95% confidence interval.

**Results:**

Forty-three epidemiological studies, four randomized trials and one meta-analysis were identified. The overall quality of these studies was evaluated as good or moderate. While some evidence suggests that green tea has beneficial effects on gastrointestinal cancers, the findings are not consistent.

**Conclusion:**

Green tea may have beneficial effects on cancer prevention. Further studies such as large and long term cohort studies and clinical trials are warranted.

## Background

Tea is one of the most popular beverages around the world. Popular in countries such as China and Japan, green tea accounts for 20% of tea consumption worldwide [1].

Green tea is derived from *Camellia sinensis*, an evergreen shrub of the Theaceae family. Unlike black tea which is fermented, green tea is produced in a non-fermented process. Green tea may be consumed in the form of a brewed beverage or capsular extract. In some countries, tea is used as dietary supplements. In China the medicinal use of green tea dated back to 4,700 years ago. Currently, there is no established recommended dose for green tea extract. Researchers examined the effects of habitually green tea drinking on cancer prevention; however, evidence has not been corroborated [2-4].

The main active ingredients of green tea include polyphenolic compounds such as epicatechin (EC), epicatechin-3-gallate (ECG), epigallocatechin (EGC) and epigallocatechin-3-gallate (EGCG), all of which may be responsible for the anti-carcinogenic and anti-mutagenic activities of green tea. Other polyphenols in green tea include flavanols and their glycosides and depsides such as cholorogenic acid, quinic acids, carotenoids, trigalloylglucose, lignin, protein, chlorophyll, minerals (aluminum or manganese, depending on the soil content), caffeine and a very small amount of methylxanthines [5].

Polyphenols in green tea were shown to be powerful antioxidants with anti-carcinogenic properties [6]. Human studies on pharmacokinetics of polyphenols in green tea were conducted [7,8]. The evidence suggests that ingested polyphenols and their metabolites play a role in the action against gastrointestinal cancers.

Many *in vitro *and *in vivo *studies demonstrated that polyphenols from green tea were anti-carcinogenic by inducing apoptosis and inhibiting cell-growth, cyclin-dependent kinase inhibitor and urokinase (an enzyme crucial for cancer growth) [1]. Probable action mechanisms include antioxidant and free-radical scavenging activity and stimulation of detoxification systems through selective induction or modification of phase I and II metabolic enzymes.

In light of growing research findings on green tea, we conducted a systematic review of randomized clinical trials and epidemiological studies to summarize the current evidence of its beneficial and harmful effects on cancer prevention in humans.

## Methods

### Databases and search strategy

We searched various electronic databases, including PubMed (1966–2008) [9], the Cochrane Library (Issue 1, 2008; CD-ROM), Chinese Biomedical Database (CD-ROM, 1978–2008). No language restriction was applied. The searches ended at March 2008. The search terms used included cancer, neoplasm, green tea, tea, *Camellia sinensis*, diet therapy, case-control study, cohort study, clinical trial, review, systematic review and meta-analysis. We also searched the following websites to identify eligible studies: NCCAM [10], the Cancer Society [11], Complementary/Integrative Medicine Education Resources (CIMER) [12], NCI Cancer Information Summaries: Complementary and Alternative Medicine [13] and the Memorial Sloan-Kettering Cancer Center [14].

### Quality assessment

As various study designs (i.e. randomized, non-randomized, prospective cohort and case-control) are included in this review, categorization after quality assessment was applied to each study [15] as follows.

#### Category A (good)

Studies in category A have the least biases and their results are considered valid. These studies are likely to consist of (1) clear description of the population, setting, interventions and comparison groups; (2) appropriate measurement of outcomes; (3) appropriate statistical and analytical methods; (4) no reporting errors; (5) less than 20 percent dropouts; (6) clear reporting of dropouts; and (7) appropriate consideration and adjustment for potential confounders.

#### Category B (fair)

Studies in category B are susceptible to some degrees of biases that are not sufficient to invalidate the results. These studies may have sub-optimal adjustments for potential confounders and may also lack certain information that is needed to assess limitations and potential problems.

#### Category C (poor)

Studies in category C have significant biases which may invalidate the results. These studies either do not consider potential confounders or do not make adjustments for them appropriately. These studies may have critical flaw in design, analysis and/or reporting, missing information and/or discrepancies in reporting.

### Inclusion criteria

We included randomized controlled trials, controlled clinical trials or observational studies including prospective cohort and case-control studies on cancer prevention of green tea oral consumption in healthy individuals or cancer patients. Systematic reviews or meta-analyses were also included. In this review, only studies in the above mentioned categories A and B were included.

### Exclusion criteria

We excluded case series, case reports, *in vitro *cell culture and animal studies. Poor quality studies (i.e. those in Category C) were also excluded.

### Data abstraction and analysis

Relative risk (RR) or hazard ratio (HR) was used for clinical trials or cohort studies, while odds ratio (OR) was used for case-control studies. The above effect estimation was presented with 95% confidence interval (95% CI).

Due to the heterogeneity of study designs, settings, interventions and outcomes, we did not conduct a meta-analysis.

## Results

A Cochrane systematic review on green tea for cancer (protocol stage) published in 2004 does not include any studies [16]. This review identified 48 clinical studies investigating the consumption of green tea and its association with the risk of developing cancer, including 42 epidemiological studies [17-58], one phase I trial [59], four randomized trials [60-63] and one meta-analysis [64] (Additional file 1). Twenty-two and 20 of the 42 epidemiological studies were cohort studies and case-control studies respectively, among which two studies [26,43] reported data from four cohort studies and one cohort study [17,18] and one case-control study [41,42] were reported twice. All studies were published between 1984 and 2008; 44 studies were published in international journals, three in Chinese [28,32,56] and one in Japanese [38].

Participants in the included studies ranged from healthy individuals, pre-cancer patients to cancer patients. Several studies reported various cancers in their cases or cohorts, but in this review these data were presented separately in relevant categories. Average sample size for cohort studies was 31,798 (from 52 to 102,137 per study), 1,099 for case-control studies (from 213 to 3,818 per study) and 182 for randomized trials (from 60 to 400 per trial). Decaffeinated green tea, tea polyphenols (500 mg or 1000 mg) or catechins was used for 3–12 months in randomized trials, whereas regular drinking tea was used for a fixed period or lifelong time in epidemiological studies. The outcomes reported from randomized trials included 8-hydroxydeoxyguanosine (8-OHdG) in urine (an indicator for oxidative DNA damage) [60,62], histopathological examination [61] and incidence of cancer [63]. Risk of cancer development was the main outcome for epidemiological studies; a few studies reported mortality [20,23], recurrence [47,49], survival of cancer [57] and sister chromatid exchange (SCE) rates [21].

Four randomized trials were placebo-controlled, double-blinded (three) and of acceptable methodological quality in terms of randomization, blinding and reporting of the studies. According to the generic quality grading for all included studies, 29 studies (60%) were evaluated as good (A) and the remaining as fair (B).

### Cancer prevention

#### Caner in general (5 studies)

A cohort study of 8,552 people with nine years of follow-up showed a negative association of green tea consumption with cancer incidence, especially among Japanese women drinking more than ten cups a day (RR 0.59; 95% CI, 0.35–0.98) [17,18]. However, a larger cohort study with 38,540 people in Japan did not show an association between green tea consumption and sum incidence of all cancers (RR 1.0; 95% CI, 0.91–1.1 for those drinking two to four times per day; RR 0.98; 95%CI, 0.88–1.1 for those drinking five times or more per day, both compared with those drinking one time or less per day) [19]. Another large cohort study found no significant benefit in terms of cancer mortality among a total of 1,134 cancer patients who consumed green tea and those who did not [20]. A randomized trial comparing green tea, black tea with water in 143 heavy smokers found significant decrease in 8-OHdG levels after a 4-month intervention [60]. A prospective cohort study in 52 male smokers demonstrated that drinking green tea inhibited cigarette-induced increase in sister chromatid exchange rates [21].

#### Oral and esophageal cancer (8 studies)

A prospective cohort study followed 20,550 men and 29,671 women for an average of 10.3 years and estimated the HRs (95% CI) in oral cancer [22]. For women, the HRs (95% CI) were 0.51 (0.10–2.68), 0.60 (0.17–2.10) and 0.31 (0.09–1.07) for green tea consumption of one to two, three to four and five or more cups per day respectively, compared with those who drank less than one cup per day (*P *for trend, 0.08). For men, no trend was observed.

Inconsistent findings exist in case-control and cohort studies on green tea drinking and esophageal cancer [23-28]. Two population-based case-control studies of 3,049 subjects found a protective effect of green tea drinking against esophageal cancer among women [24,25], while another case-control study of 1,043 subjects showed 39% decrease of risk of esophageal cancer among alcoholic drinkers and 31% decrease among smokers [28]. The tea polyphenol epigallocatechin in urine was inversely associated with cancer risk when the data of gastric and esophageal cancer sub-sites were combined [27], indicating protective effect of green tea. However, in terms of death, a retrospective cohort and a pooled analysis of two cohort studies with a total of 26,723 subjects demonstrated a positive association (HR 1.67; 95% CI, 0.89–3.16) (*P *for trend, 0.04) of drinking green tea and mortality of esophageal cancer among men [23,26]. A randomized controlled trial with 400 participants who were pathologically confirmed to have esophageal precancerous lesions did not show benefit of decaffeinated green tea for alleviating precancerous lesions or abnormal cell proliferation compared with calcium as placebo [61].

#### Stomach cancer (11 studies)

Seven epidemiological studies (one cohort and six case-control studies) with a total number of 77,777 subjects showed inverse association of green tea consumption (urine polyphenol epigallocatechin in one study) and the risk reduction of stomach cancer [27,28,30,32,34,36,37]. The cohort study with 72,943 subjects showed benefit for women who consumed five or more cups of green tea per day (RR 0.51; 95% CI, 0.30–0.86) compared with one cup per day [30]. However, four studies including two cohort studies with 102,179 subjects did not show an inverse association of green tea consumption and risk reduction of stomach cancer or cancer-caused death [29,30,33,35].

#### Pancreatic cancer (4 studies)

Two case-control studies (in three publications) with 522 pancreatic cancer patients and 1,694 controls showed an inverse association of drinking green tea and the risk of pancreatic cancer [38,41,42]. By contrast, a hospital-based case-control study of 124 patients and 124 controls demonstrated a positive association of drinking five cups or more green tea per day and pancreatic cancer [39]. A population-based cohort study involving 102,137 participants with 11 years of follow-up did not find any association of the risk of pancreatic cancer and drinking green tea [40].

#### Liver cancer (2 studies)

A population-based case-control study involving 204 patients and 415 controls reported that drinking green tea reduced the risk of liver cancer by 78% (OR 0.25; 95% CI, 0.11–0.57) among alcoholic drinkers and by 43% among smokers [28]. A randomized, double-blinded, placebo-controlled trial in 124 individuals with sero-positive HBsAg and aflatoxin-albumin adducts showed a significant decrease of 8-hydroxydeoxyguanosine after three months of green tea polyphenols intake [62].

#### Biliary ducts cancer (1 study)

Statistical analysis showed that green tea consumption was positively associated with the mortality of biliary duct cancer in a retrospective cohort study (*P *≤ 0.01) [23].

#### Colorectal cancer (6 studies)

Two case-control studies (in three publications) involving 2,036 patients with colorectal cancer and 2,130 controls found that drinking green tea reduced the risk of colorectal cancer [41,42,44]. Gender difference was observed in two cohort studies [45,46]. A prospective cohort study that followed over 60,000 subjects for an average of 8.9 years found no statistically significant difference between green tea drinkers and non-drinkers (RR 1.12; 95% CI, 0.97–1.29) [45]. Another cohort study with six years of follow-up on 69,710 women found significant dose-response relationship (RR 0.63; 95% CI, 0.45–0.88) between regular and non-regular green tea drinkers [46]. By contrast, a pooled analysis from two cohort studies on over 60,000 subjects showed no association between drinking green tea and a lower risk of colorectal cancer [43].

#### Breast cancer (5 studies)

In a meta-analysis of two prospective cohorts of 35,004 Japanese women [64], green tea intake was not associated with a lower risk of breast cancer (222 cases); and the multivariate RR for women drinking more than five cups of green tea was 0.84 (95% CI, -0.57–1.24; *P *= 0.69) compared with those drinking less than one cup per day. One case-control study showed significantly reduced risk of breast cancer by regular drinking a large amount of green tea [50]. However, a cohort study did not find an association of green tea intake with lower risk of breast cancer [48]. Another two cohort studies showed reduced recurrence of breast cancer among patients at stage I and II with high consumption of green tea (more than three cups per day) [47,49].

#### Lung cancer (4 studies)

A randomized trial compared green tea and black tea with water in 143 heavy smokers for 4 months [60]. The content of 8-OHdG in urine was reduced among the subjects in green tea group, but not in black tea or water groups. A cohort study of 52 male adults, found that green tea drinking blocked cigarette-induced increase in sister chromatid exchange rates, suggesting potential protection against lung cancer [21]. Consumption of green tea was found to be associated with a reduced risk of lung cancer among non-smoking women in one case-control study [51]. A phase I dose finding study showed the maximum tolerated dose of green tea extract as 3 g/m^2 ^per day in patients with advanced lung cancer. The dose-limiting toxicities were diarrhea, nausea and hypertension [59].

#### Prostate cancer (4 studies)

A double-blind, placebo-controlled trial testing green tea catechins (600 mg per day for one year) significantly reduced the incidence of prostate cancer in a group of 60 volunteers with high-grade prostate intraepithelial neoplasia; no significant adverse effect was reported [63]. A case-control study found prostate cancer risk declined with increasing frequency, duration and quantity of green tea consumption [53]. A cohort study in 19,561 Japanese men showed that green tea intake was not associated with a lower risk of prostate cancer (HR 0.85; 95% CI, 0.50–1.43) between men drinking five or more cups and less than one cup per day [54]. However, another recent cohort study with a larger sample size (*n *= 49,920) in Japan suggested that green tea was associated with a decreased risk of advanced prostate cancer (RR 0.52; 95% CI, 0.28–0.96) in men drinking five or more cups compared with those drinking less than one cup per day [55].

#### Urinary bladder cancer (1 study and 1 ongoing trial)

The risk of urinary bladder cancer was significantly reduced in women who consumed *matcha *(a powdered green tea) in a case-control study (*n *= 882) [52]. A phase II randomized, double-blind, placebo-controlled, multi-center trial (*n *= 330) is currently carried out in the United States [65].

#### Endometrial cancer (1 study)

A population-based case-control study (*n *= 2082) suggested that regular green tea drinking reduced the risk of endometrial cancer (OR 0.74; 95% CI, 0.54–1.01) in pre-menopausal women [56].

#### Ovarian cancer (1 study)

A cohort study (*n *= 254) suggested that increasing the post-diagnosis consumption of green tea may boost the survival of patients of epithelial ovarian cancer [57].

#### Adult leukemia (1 study)

A case-control study (*n *= 217) demonstrated statistical association (OR 0.51; 95% CI 0.27–0.96) between higher intake of green tea and reduced risk of adult leukemia in a dose-response manner [58].

### Safety of green tea

Green tea, as a popularly consumed beverage, is relatively non-toxic [66]. Phase I trial in 17 patients with advanced lung cancer showed that the maximum tolerated dose of green tea extract was 3 g/m^2 ^per day [59]. No severe adverse effects have been reported in association with the medicinal use of green tea [67]. Consumption of high doses of green tea or green tea extract (i.e. 5–6 litters per day) may cause nausea, vomiting, abdominal bloating/pain, dyspepsia, flatulence and diarrhea [59,67]. Excessive consumption of caffeine from green tea may also cause central nervous system stimulation such as dizziness, insomnia, tremors, restlessness, confusion, diuresis (i.e. increasing urine output), heart rate irregularities and psychomotor agitation [67].

Human studies did not show severe adverse effects among volunteers who took 15 tablets of green tea per day (i.e. 2.25 g green tea extracts, 337.5 mg EGCG and 135 mg caffeine) for 6 months [68,69]. A randomized, placebo-controlled trial (*n *= 40) found no adverse effect in healthy individuals who took green tea polyphenols in the amount equivalent to the EGCG content in 8–16 cups of green tea once a day or twice a day in divided doses for four weeks [69].

## Discussion

Current research findings are not sufficient to validate the effects of green tea on cancer prevention as most evidence coming from cohort (grade III) and case-control studies (grade IV) is not consistent. As tea drinking is common in many populations, it is difficult to randomize subjects into drinking groups or non-drinking groups. For the same reason, epidemiological studies are still important in this field.

This review of randomized trials and epidemiological studies shows the current evidence in green tea and cancer prevention. More than half of the studies (58%) suggest that long-term consumption of green tea may reduce the risk of certain types of cancer, in particular gastrointestinal cancers, such as esophageal, stomach, pancreatic, liver and colorectal cancer. Stratified analysis suggests that women benefit more than men from green tea drinking. However, the beneficial effects are not consistent across all studies. The interpretation of these findings is a challenge due to the significantly heterogeneous study designs, settings, populations, exposures, comparisons, outcome measures and potential publication biases. The heterogeneity hinders meaningful meta-analysis despite the large number of studies covered in this systematic review. The discrepancies in the findings may be due to the following factors: (1) participants in terms of health status, family history of cancer, age, gender, ethnic and other lifestyle confounders such as smoking or alcohol drinking; (2) definitions of green tea consumption, e.g. frequency, duration, quantity of green tea and the quality of green tea products; and (3) study designs and trial settings.

This review shows that the overall evidence for protective effects of green tea against cancer is inconclusive. Therefore, further prospective cohort studies and clinical trials are warranted. Adequate sample size, better descriptions of populations and/or clear definitions of green tea consumption may be required for conclusive studies.

Moderate consumption of green tea (3–9 cups per day) is generally safe. People with known allergy/hypersensitivity to caffeine or tannin should avoid green tea. In general, the stimulatory effect of green tea is considerably less than that of coffee [66]. However, pregnant women, nursing mothers and patients with cardiac conditions are advised to avoid or limit their intake of green tea to two cups per day [68].

## Conclusion

Great efforts have been made to show the beneficial effects of green tea consumption on various cancers. Some epidemiological studies demonstrated protective effects of green tea consumption on gastrointestinal, breast, lung and prostate cancer. However, these findings have not been confirmed by other studies covered in this review. Future prospective studies are therefore warranted.

## Competing interests

The authors declare that they have no competing interests.

## Authors' contributions

JPL conceived and drafted this article. JMX and YTF helped select studies and abstracted data. JPL validated the process and conducted data analysis. All authors contributed to the writing and approved the final version of the manuscript.

## Supplementary Material

Additional file 1 **Summary of the included studies on green tea for cancer prevention.** The table provides summarized information of the studies included in the present systematic review, including names of authors, location, study design, study quality, type of cancer, population and main findings. Click here for file
