# Supplementary material for: Green tea (Camellia sinensis) and cancer prevention: a systematic review of randomized trials and epidemiological studies
Source: Chin Med. 2008 Oct 22;3:12. doi: 10.1186/1749-8546-3-12 (PMC2577676; doi:10.1186/1749-8546-3-12)
Supplement: Additional file 1 — Summary of the included studies on green tea for cancer prevention. The table provides summarized information of the studies included in the present systematic review, including names of authors, location, study design, study quality, type of cancer, population and main findings. [file 1749-8546-3-12-S1.doc]

**Summary of the included studies on green tea for cancer prevention**

| **Author (location)** | **Study design** | **Study quality*** | **Type of cancer** | **Population** | **Main findings** | | **Reference** |
| --- | --- | --- | --- | --- | --- | --- | --- |
| ***All kinds of cancer*** | | | | | | |  |
| Imai 1997  and Nakachi 2000 (Japan) | prospective cohort study | A | all cancer | 8,552 individuals over 40 years in Saitama Prefecture and 384 cases of cancer identified by 9 years of follow-up | There was a negative association between green tea consumption and cancer incidence, especially among females drinking >10 cups/day (RR 0.57; 95% CI, 0.33-0.98). A significant delay in cancer onset was associated with increased consumption of green tea. Green tea has a potentially preventive effect against cancer among humans. | | [17,18] |
| Nagano 2001 (Japan) | prospective cohort study | B | all cancer | 38,540 Japanese | Green tea consumption was virtually unrelated to incidence of cancers. RRs for all cancers for those consuming green tea 2-4 times/day and ≥5 times/day were 1.0 (95% CI, 0.91-1.1) and 0.98 (0.88-1.1) respectively, as compared with those consuming ≤1 time/day. No evidence supports relationship of green tea consumption and reduced cancer risks. | | [19] |
| Kuriyama 2006 (Japan) | prospective cohort study | A | all cancer | 40,530 Japanese adults aged 40-79 years | During over 7 years of follow-up, 1134 participants died of cancer. HRs of cancer mortality were not significantly different from 1.00 in all green tea categories (1-2 cups, 3-4 cups, ≥5 cups/day) compared with the lowest consumption (≤1 cup/day). | | [20] |
| Hakim 2003 (USA) | randomized controlled trial | A | smokers | 143 heavy smokers randomized to receive black, green tea or water | 8-hydroxydeoxyguanosine (8-OHdG) in urine was measured as an indicator of oxidative DNA damage. After four months, 8-OHdG was decreased significantly in smokers of drinking decaffeinated green tea (-31%, *P*=0.002) after adjustment for baseline measurements and other potential confounders, but no change was observed among smokers in the black tea group. | | [60] |
| Shim JS 1995 (Japan) | prospective cohort with internal controls | B | cigarette smokers | 52 male subjects | Sister chromatid exchange (SCE) rates were elevated significantly in smokers (mean 9.46; SD 0.46) versus non-smokers (mean 7.03; SD 0.33). SCE rates in smokers (mean 7.94; SD0.31) who consumed green tea 2-3 cups/day for 6 months were comparable to non-smokers, suggesting protecting effect of green tea on cigarette-induced increase in SCE frequency. | | [21] |
| ***Digestive cancer*** | | | | | | |  |
| Ide 2007 (Japan) | prospective cohort study | A | oral cancer | 20,550 men and 29,671 women aged 40-79 years with an average of 10.3 years follow up | | The incidence of oral cancer was 0.74‰ (37 out of 50221). In women, the HRs of oral cancer for green tea consumption of 1-2, 3-4 and ≥5 cups/day were 0.51 (95% CI, 0.10-2.68), 0.60 (95% CI, 0.17-2.10) and 0.31 (95% CI, 0.09-1.07) respectively, compared with those who drank ≤1 cup/day. In men, no such trend was observed. | [22] |
| Hara 1984 (Japan) | retrospective cohort study | B | digestive tract | unspecified males in 46 prefectures | | Statistical analyses on 26 patterns of food consumption and mortality of six digestive tract cancers were performed. It showed that green tea was positively associated with esophageal cancer and biliary duct cancer (*P*≤0.01). | [23] |
| Wang LD 2002 (China) | randomized controlled trial | B | esophageal precancerous lesions (EPL) | 400 participants with pathologically confirmed EPL randomly divided into decaffeinated green tea (DGT) group and placebo (calcium) group. | | DGT (5mg/day for 12 months) did not show beneficial effects in alleviating EPL or abnormal cell proliferation compared with calcium (1200mg/day for 11 months) through esophageal histopathological examination. | [61] |
| Gao YT 1994 (China) | population-based, case-control study | A | esophagus | 734 out of 902 patients interviewed had pathologically confirmed cancer; and 1552 controls | | After adjustment for age, gender and other known confounders, a protective effect of green tea drinking on esophageal cancer was observed among women (OR 0.50; 95% CI, 0.30-0.83) and this risk decreased as tea consumption increased. | [24] |
| Wang 2007 (China) | population-based, case-control study | A | esophageal squamous cell carcinoma | 355 histologically confirmed esophageal cancer cases and 408 controls matched by sex and age | | Green tea drinking showed a protective effect in women (OR 0.26; 95% CI, 0.07-0.94). | [25] |
| Ishikawa 2006 (Japan) | pooled analysis of two prospective cohort studies | B | Esophageal cancer | 9,008 men in cohort 1 and 17,715 men in cohort 2, aged ≥40 years, without history of cancer | | 38 cases with esophageal cancer in cohort 1 (9.0 years of follow-up) and 40 cases in cohort 2 (7.6 years of follow-up) were identified. Compared with men who had never drunk green tea, the pooled multivariate HR was 1.67 (95% CI, 0.89-3.16) (*P* for trend=0.04) for men who were currently drinking ≥5 cups/day green tea. Green tea consumption was significantly associated with an increased risk of esophageal cancer. | [26] |
| Sun 2002  (China) | nested case-control study | A | esophagus, stomach | 190 cases of gastric cancer, 42 cases of esophageal cancer and 772 controls | | Tea polyphenol epigallocatechin (EGC) in urine showed a statistically significant inverse association with gastric cancer (OR 0.52; 95% CI, 0.28-0.97) after adjustment for *H. pylori* seropositivity, smoking, alcohol drinking and level of serum carotenes. Similar tea polyphenol-cancer risk associations were observed when the data of gastric and esophageal cancer were combined. | [27] |
| Mu LN 2003 (China) | population-based, case-control study | B | esophagus, stomach, liver | 218, 206 and 204 for esophageal, stomach and liver cancer patients respectively and 415 controls | | Drinking green tea decreased risk of developing stomach, liver and esophageal cancer by 81%, 78% and 39% respectively among alcoholic drinkers; by 16%, 43% and 31% respectively among smokers. For the risk decrease of stomach and liver cancer for alcoholic drinkers, ORs were 0.23 (95% CI, 0.10-0.55) and 0.25 (0.11-0.57) respectively. | [28] |
| Hoshiyama 2002 (Japan) | prospective cohort study | A | stomach | 30,370 men and 42,481 women aged 40-79 years | | After adjustment for age, smoking status, history of peptic ulcer and family history of stomach cancer along with certain dietary elements, the risks associated with drinking 1-2 cups, 3-4 cups, 5-9 cups and ≥10 cups/day were 1.6 (95% CI, 0.9-2.9), 1.1 (0.6-1.9), 1.0 (0.5-2.0) and 1.0 (0.5-2.0) respectively, compared to those of drinking <1 cup daily. There was no inverse association between green tea consumption and the death risk of stomach cancer. | [29] |
| Sasazuki 2004 (Japan) | population-based, prospective cohort study | A | stomach | 72,943 followed for 10 years and 892 gastric cancer cases were identified | | An inverse association between green tea consumption and distal gastric cancer was observed among women (RR 0.51; 95% CI, 0.30-0.86) in the highest category of green tea consumption (≥5 cups/day versus ≤1 cup/day). | [30] |
| Tsubono 2001 (Japan) | population-based, prospective cohort study | A | stomach | 26,311 residents in Miyagi Prefecture followed and 419 cases of gastric cancer identified | | After adjustment for age, smoking, history of peptic ulcer, alcohol, other dietary elements and type of health insurance, the RRs associated with drinking 1-2 cups, 3-4 cups, ≥5 cups/day, as compared with <1 cup/day, were 1.1 (95% CI, 0.8-1.6), 1.0 (0.7-1.4) and 1.2 (0.9-1.6) respectively. There was no association between green tea consumption and the risk of stomach cancer. | [31] |
| Yu SZ 2001 (China) | population-based, case-control study | A | stomach | 143 cases of stomach cancer and 433 controls | | Drinking green tea decreased risk of developing stomach cancer by 54% (OR 0.46; 95% CI, 0.22-0.96). | [32] |
| Hoshiyama 2004 (Japan) | nested case-control study | A | stomach | 157 incident cases and 285 controls aged 40-79 years | | After adjustment for age, smoking status, *H. pylori* infection, history of peptic ulcer and family history of stomach cancer along with certain dietary elements, the risks associated with drinking 1-2 cups, 3-4 cups, 5-9 cups and ≥10 cups/day were 1.3 (95% CI, 0.6-2.8), 1.0 (0.5-1.9), 0.8 (0.4-1.6) and 1.2 (0.6-2.5) respectively. Green tea consumption showed no protective effect against stomach cancer. | [33] |
| Kono 1988 (Japan) | case-control study | B | stomach | 139 cases and 278 controls | | A decreased risk of stomach cancer was noted among those with high consumption of green tea (≥10 cups/day) (*P*<0.05). | [34] |
| Ji 1996 (China) | case-control study | B | stomach | 1124 cases of stomach cancer and 1451 controls | | Green tea drinking was not statistically associated with risk of stomach cancer no matter anatomic sub-site (OR 0.77; 95% CI, 0.52-1.13 among female heavy drinkers; OR 0.76; 95% CI, 0.55-1.27 among male heavy drinkers). | [35] |
| Yu GP 1995 (China) | case-control | A | stomach | 711 cases of primary stomach cancer aged under 80 years; 711 controls matched with age and gender | | Comparing drinkers of green tea and nondrinkers (OR 0.71; 95% CI, 0.54-0.93), suggesting green tea consumption associated with lower risk of stomach cancer. | [36] |
| Tajima 1985 (Japan) | case-control study | B | stomach, colon | 93 cases with stomach cancer, 93 cases with colorectal cancers and 186 controls | | Consumption of green tea decreased risk of stomach, colon and rectum cancer (OR 0.64, 0.97, 0.91 respectively) when compared with tea drinkers with non-drinkers. | [37] |
| Goto 1990 (Japan) | case-control study | B | pancreas | 71 cases and 142 controls | | Significantly decreased risks of pancreatic cancer were associated with consumption of raw vegetables and green tea. | [38] |
| Mizuno 1992 (Japan) | hospital-based, case-control | B | pancreas | 124 cases and 124 controls | | The risk of developing pancreatic cancer was positively associated with drinking green tea ≥5 cups/day. | [39] |
| Luo 2007 (Japan) | population-based cohort study | A | pancreas | 102,137 participants followed for average of 11 years | | The incidence of pancreatic cancer was 0.23% (233/102137). Overall, the risk of pancreatic cancer was not associated with green tea. | [40] |
| Ji 1997  Dai Q 1996  (China) | population-based, case-control study | B | pancreas, colon, rectum | 931 cases of colon cancer, 884 cases of rectum cancer, 451 cases of pancreas cancer; and 1,552 controls | | Inverse association with each cancer was observed with increasing amount of green tea consumption, with the strongest trends for rectal and pancreatic cancers. For men, compared with non-regular tea drinkers, ORs among those in the highest consumption (>300 g/month) were 0.82 for colon cancer, 0.72 for rectal cancer and 0.63 for pancreatic cancer, *P*=0.38, 0.04 and 0.04 respectively. For women, the respective ORs for the highest consumption (>200 g/month) were 0.67, 0.57 and 0.53, with *P*=0.07, 0.001 and 0.008 respectively. | [41],[42] |
| Suzuki 2005 (Japan) | pooled analysis of 2 cohort studies | A | colorectal cancer | 26,311 (1^st^ cohort) and 39,604 (2^nd^ cohort) Japanese with 7 to 9 years of follow-up | | 305 colon and 211 rectal cancers were identified in the two cohorts. Pooled HRs for colon cancer associated with drinking green tea 1-2, 3-4 and ≥5 cups/day were 1.06 (95% CI, 0.74-1.52), 1.10 (0.78-1.55), 0.97 (0.70-1.35) respectively; HRs for rectal cancer were 0.85 (0.56-1.29), 0.70 (0.45-1.08), 0.85 (0.58-1.23) respectively, compared to <1 cup/day. No association was found between green tea consumption and lower risk of colorectal cancer. | [43] |
| Kato 1990 (Japan) | case-control study | B | colon, rectum | 221 cases of colorectal cancer and 578 controls | | Daily intake of green tea was inversely associated with distal colon and rectal adenomas (OR 0.61; 95% CI, 0.40-0.92, 0.61, 0.41-0.91 respectively). | [44] |
| Sun 2007 (Singapore) | prospective cohort study | A | colorectal cancer | >60,000 men and women with an average of 8.9 years follow-up | | There was no statistically significant difference between green tea drinkers and non-drinkers (RR 1.12; 95% CI, 0.97-1.29). In men, the association of green tea and colorectal cancer was noted mainly in those with advanced cancer (Duke C or D) (RR 1.53; 95% CI, 1.19-1.97). | [45] |
| Yang 2007 (China) | prospective cohort study | A | colorectal cancer | 69,710 Chinese women aged 40-70 years | | 256 cases of colorectal cancer were identified during 6 years of follow up (incidence rate 0.37%). RR of colorectal cancer was 0.63 (95% CI, 0.45-0.88) for women with regular green tea drinking compared with non-regular tea drinking. A significant dose-response relationship was found for both the amount of tea consumed and duration in years of lifetime tea consumption. | [46] |
| Luo 2006 (USA) | randomized, double-blinded, placebo-controlled trial | A | liver | 124 individuals with sero-positive for both HBsAg and aflatoxin-albumin adducts | | Daily taking green tea polyphenols (at 500mg or 1000mg) for 3 months decreased significantly the level of 8-OHdG (an indicator for oxidative DNA damage) compared with placebo. | [62] |
| ***Breast cancer*** | | | | | | |  |
| Suzuki 2004 (Japan) | Meta-analysis of 2 cohorts | B | breast | 222 cases out of 35,004 women followed | Green tea drinking was not associated with a lower risk of breast cancer and the multivariate RR for women drinking ≥5 cups/day compared with <1 cup/day was 0.84 (95% CI, -0.57-1.24, *P*=0.69). | | [64] |
| Inoue M 2001 (Japan) | hospital-based, cohort study | A | breast | 1160 new surgical cases of female invasive breast cancers | Decreased HR for recurrence in stage I was observed with green tea consumption ≥3 cups/day (HR 0.43, 95% CI, 0.22-0.84), suggesting that regular green tea drinking may have protective effect against recurrence of breast cancer in early stage cases. | | [47] |
| Key 1999 (Japan) | prospective cohort study | A | breast | 427 cases of primary breast cancer out of 34,759 women | Green tea drinking was not associated with a lower risk of breast cancer and the RRs for women drinking ≤1 cup, 2-4 cups, ≥5 cups/day were 1.0, 1.02 (95% CI, 0.76-1.36) and 0.86 (0.62-1.21) respectively. | | [48] |
| Nakachi 1998 (Japan) | cohort study | A | breast | 472 patients with stage I, II, III breast cancer | Increased consumption of green tea was correlated with decreased recurrence of stage I and II breast cancer (*P*<0.05 for crude disease-free survival); the recurrence rate was 16.7% or 24.3% among those consuming ≥5 cups or ≤4 cups/day respectively, in a 7-year follow-up and the RR of recurrence was 0.56 (95% CI, 0.35-0.91) after adjustment for other lifestyle factors. There was no improvement in prognosis in stage III breast cancer. | | [49] |
| Wu 2003 (USA) | population-based, case-control study | A | breast | 501 breast cancer patients with Chinese, Japanese or Filipino origin and 594 controls | Green tea drinkers showed a significantly reduced risk of breast cancer and this was maintained after adjusting for age, specific Asian ethnicity, birthplace, age at menarche, parity, menopausal status, use of menopausal hormones, body size and intake of total calories and black tea. Compared to women with non-regular drinking, there was a significant trend of decreasing risk with increasing amount of green tea drinking, adjusted ORs 1.00, 0.71 (95% CI, 0.51-0.99) and 0.53 (0.35-0.78) respectively, in association with no, 0-85.7 and >85.7ml of green tea/day. The significant inverse association between risk of breast cancer and green tea remained after further adjustment for other potential confounders, including smoking; alcohol, coffee and black tea intake; family history of breast cancer; physical activity; and intake of soy and dark green vegetables. | | [50] |
| ***Lung cancer*** | | | | | | |  |
| Laurie 2005 (USA) | Phase I dose finding study | A | lung | 17 patients with advanced lung cancer | The maximum tolerated dose of green tea extract was 3g/m^2^ per day; and the dose-limiting toxicities were diarrhea, nausea and hypertension. | | [59] |
| Zhong L 2001 (China) | case-control | A | lung | 649 women with primary lung cancer; 675 women matched to age | Among non-smoking women, consumption of green tea was associated with a reduced risk of lung cancer (OR 0.65; 95% CI, 0.45-0.93) and the risks decreased with increasing consumption. | | [51] |
| ***Urinary or reproductive cancer and others*** | | | | | | |  |
| Ohno 1985 (Japan) | population-based, case-control study | B | urinary bladder | 293 patients and 589 controls | Reduced risk of significance (crude RR 0.45; 95% CI, 0.22-0.92) was found for intake of black tea and for females who consumed matcha (powdered green tea). | | [52] |
| Jian 2004 (China) | case-control study | A | prostate | 130 incident patients and 274 controls | Among the cases, 55% were green tea drinkers compared to 80% for the controls. The prostate cancer risk declined with increasing frequency, duration and quantity of green tea consumption and the adjusted OR were 0.28 (95% CI, 0.17-0.47) for tea drinking, 0.12 (0.06-0.26) for drinking tea over 40 years, 0.09 (0.04-0.21) for those consuming >1.5kg of tea leaves yearly and 0.27 (0.15-0.48) for those drinking >3 cups/day compared to non-tea drinkers. | | [53] |
| Bettuzzi 2006 (Italy) | double-blind, placebo-controlled trial | A | prostate | 60 volunteers with high-grade prostate intraepithelial neoplasia | Oral administration of green tea catechins (600mg/d) for 1 year showed significantly lower incidence of prostate cancer (biopsy confirmed diagnosis) compared with placebo (1/30 versus 9/30, *P*<0.01). | | [63] |
| Kikuchi 2006 (Japan) | prospective cohort study | B | prostate | 19,561 Japanese men aged 40-79 years | 110 cases with prostate cancer were identified during 7 years of follow up. Green tea intake was not associated with a lower risk of prostate cancer and the multivariate HR for men drinking ≥5 cups/day compared with <1 cup/day was 0.85 (95% CI, 0.50-1.43, trend *P*=0.81). | | [54] |
| Kurahashi 2008 (Japan) | cohort study | A | prostate | 49,920 men aged 40-69 years | The incidence of prostate cancer in the cohort was 0.81% (404 out of 49920). Green tea was associated with a decrease risk of advanced prostate cancer (RR 0.52; 95% CI, 0.28-0.96; *P*=0.01) for men drinking ≥5 cups/day compared with <1 cup/day. However, green tea was not associated with localized prostate cancer. | | [55] |
| Gao 2005 (China) | population-based, case-control study | A | endometrial cancer | 995 patients with endometrial cancer and 1087 controls | Compared to non-tea drinkers, regular green tea drinkers had reduced risk of endometrial cancer (OR 0.74; 95% CI, 0.54-1.01) in pre-menopausal women. There was a weak but inverse association in tea drinking and endometrial cancer risk. | | [56] |
| Zhang M 2004 (China) | cohort study | A | ovarian | 254 Chinese patients confirmed epithelial ovarian cancer | Increasing the consumption of green tea post-diagnosis may enhance epithelial ovarian cancer survival. | | [57] |
| Zhang M 2008 (China) | case-control study | B | adult leukemia | 107 adults with leukemia and 110 orthopedic controls | Higher intake of green tea was associated with a reduced risk of adult leukemia (OR 0.51; 95% CI, 0.27-0.96; *P*=0.04) and with significant dose-response relationships (longer duration, higher quantity and frequency of intake). | | [58] |

*Study quality is categorized as A (good) or B (fair).

RR: relative risk, OR: odds ratio, HR: hazard ratio, CI: confidence interval
